# Preferential phosphatidylglycerol synthesis via phosphorus supply through rRNA degradation in the cyanobacterium, *Synechocystis* sp. PCC 6803, under phosphate-starved conditions

**DOI:** 10.3389/fpls.2024.1335085

**Published:** 2024-01-29

**Authors:** Tatsunori Hiyoshi, Megumi Haga, Norihiro Sato

**Affiliations:** School of Life Sciences, Tokyo University of Pharmacy and Life Sciences, Hachioji, Japan

**Keywords:** cyanobacteria, phosphatidylglycerol, phosphorus starvation, rRNA, *Synechocystis*

## Abstract

Photosynthetic organisms often encounter phosphorus (P) limitation in natural habitats. When faced with P limitation, seed plants degrade nucleic acids and extra-plastid phospholipids to remobilize P, thereby enhancing their internal-P utilization efficiency. Although prokaryotic and eukaryotic photosynthetic organisms decrease the content of phosphatidylglycerol (PG) under P-limited conditions, it remains unclear whether PG is degraded for P remobilization. Moreover, information is limited on internal-P remobilization in photosynthetic microbes. This study investigates internal-P remobilization under P-starvation (-P) conditions in a cyanobacterium, *Synechocystis* sp. PCC 6803, focusing on PG and nucleic acids. Our results reveal that the PG content increases by more than double in the -P culture, indicating preferential PG synthesis among cellular P compounds. Simultaneously, the faster increases of glycolipids counteract this PG increase, which decreases the PG proportion in total lipids. Two genes, *glpD* and *plsX*, contribute to the synthesis of diacylglycerol moieties in glycerolipids, with *glpD* also responsible for the polar head group synthesis in PG. The mRNA levels of both *glpD* and *plsX* are upregulated during -P, which would cause the preferential metabolic flow of their P-containing substrates toward glycerolipid synthesis, particularly PG synthesis. Meanwhile, we find that RNA accounts for 62% of cellular P, and that rRNA species, which makes up the majority of RNA, are degraded under -P conditions to less than 30% of their initial levels. These findings emphasize the importance of PG in -P-acclimating cell growth and the role of rRNA as a significant internal-P source for P remobilization, including preferential PG synthesis.

## Introduction

Phosphorus (P), along with nitrogen (N) and sulfur (S), forms organic carbon compounds through covalent bonds. P-compounds include nucleic acids, phospholipids, and a variety of intermediate metabolites. Together with N and S, P is defined as a macronutrient in photosynthetic organisms (Kumar et al., 2021). Photosynthetic organisms often face nutrient deficiency stresses, with phosphate limitation, or P-limitation, being a common stressor. A series of P-limitation acclimation strategies, including improvements in external phosphate (Pi) acquisition ability and internal P use efficiency (PUE), have been developed in photosynthetic organisms (Plaxton and Tran, 2011; Heuer et al., 2017). Intriguingly, photosynthetic organisms exhibit milder symptoms in physiological processes with P-starvation (-P) compared to those with N or S-starvation, at least during the short-term stresses (Mao et al., 2018; Hirai et al., 2019; Teixeira et al., 2021). Notably, in a cyanobacterium, *Synechocystis* sp. PCC 6803, -P had a much smaller impact on cell growth compared to N or S-starvation (an 8-fold increase from the initial level with -P and a 2.5-fold increase with -N or -S, c.f., an 11-fold increase under nutrient-replete conditions; Hirai et al., 2019). The features of these photosynthetic organisms demonstrated that the utilization efficiency of a nutrient under starvation stress, relative to that under repletion, was much higher for P than for N or S.

The mechanism by which photosynthetic organisms establish high PUE under -P conditions have been investigated at the biochemical and molecular levels. In seed plants, PUE is improved through P remobilization between organs or tissues, such as from old leaves to young ones and growing roots (Rausch and Bucher, 2002). Nucleic acids generally make up 40-60% of organic P, with rRNA representing approximately 80% of nucleic-acid P (Veneklaas et al., 2012; Smith et al., 2015). Due to the abundance of rRNA and the induced expression of cytosolic RNase genes under P-limiting conditions, it is regarded that rRNA serves as a major source for P remobilization in seed plants acclimating to P-limitation stress (Młodzińska and Zboińska, 2016). Seed plants also remobilize P from chloroplast and mitochondrial DNA, which accounts for 6-9% of total nucleic acids (Takami et al., 2018). Concerning photosynthetic microbes, however, information is only fragmentary on nucleic-acid related PUE improvement: a green alga, *Chlamydomonas reinhardti* showed a decrease in the number of chloroplast nucleoids with P-limitation, with mRNA in chloroplasts enhanced in stability through the repression of the gene for PNPase, a phosphorylytic RNase (Yehudai-Resheff et al., 2007). It was therefore proposed that -P cells of *C. reinhardtii* degrade chloroplast DNA but not its RNA for P-remobilization.

Membrane lipid metabolism also plays a crucial role in increasing PUE in photosynthetic organisms. In green algae and seed plants, phosphatidylcholine (PC) and phosphatidylethanolamine (PE), major components of phospholipids that occupy 15-30% of total P, are markedly degraded in extra-plastid membranes under -P conditions through the actions of phospholipases C and/or D (Andersson et al., 2003; Cruz-Ramírez et al., 2006; Gaude et al., 2008; Nakamura et al., 2009; Oishi et al., 2022). This phospholipid losing process sequesters P from these phospholipids as an internal-P source and is accompanied by compensating increases in non-phospholipid contents. This replacement of phospholipids by non-phospholipids in extra-plastid membranes, therefore, contributes to the improvement in PUE.

In this context, it is noteworthy that -P-stress additionally induces another type of lipid remodeling for PUE improvement, which involves decreases in anionic phospholipid, phosphatidylglycerol (PG), along with increases in anionic non-phospholipid, sulfoquinovosyl diacylglycerol (SQDG), in photosynthetic bacteria, cyanobacteria, and algal and seed-plant plastids (Benning et al., 1993; Güler et al., 1996; Essigmann et al., 1998; Riekhof et al., 2003; Awai et al., 2007; Van Mooy et al., 2009; Endo et al., 2016; Murakami et al., 2018; Peng et al., 2019; Oishi et al., 2022). This lipid remodeling, maintaining not only lipid contents in photosynthetic membranes but also their charge balance for photosynthesis functionality, appears to be conserved throughout the evolution of photosynthetic organisms (Sato, 2004). The molecular mechanism underlying -P-induced increases in SQDG includes upregulation of expression levels of the genes for SQDG synthesis (*sqdB* in cyanobacteria or *UGP3*/*SQD1*/*SQD2* in algae and plants), with some of these genes found to be indispensable for maintenance of -P-acclimating growth of the organisms (Benning et al., 1993; Güler et al., 1996; Yu et al., 2002; Riekhof et al., 2003; Okazaki et al., 2009; Sato and Wada, 2009; Hung et al., 2015; Endo et al., 2016). However, the metabolic mechanism governing the decrease in PG and its physiological significance are yet to be elucidated, including whether or not PG is degraded for the acquisition of internal P.

Differing from seed plants, photosynthetic microbes, especially unicellular ones, need to carry out P remobilization in separate cells. We here investigated how cells of a unicellular cyanobactrium, *Synechocystis* sp. PCC 6803, remobilized intracellular P under -P conditions, focusing on the quantitative behavior of PG and nucleic acids. Our results demonstrated that under -P conditions, PG synthesis was promoted through the preferential incorporation of internal P, and that rRNA was degraded to provide a primary internal-P source for P remobilization.

## Materials and methods

### Cyanobacterial strains and growth conditions

*Synechocystis* sp. PCC 6803 cells were cultured at 30°C in tubes containing either a standard BG11 medium with phosphate or a modified BG11 medium in which phosphate was replaced with Cl- (Hiyoshi et al., 2021). The cultures were aerated with air and illuminated with fluorescence light (10 W·m^-2^). The optical density at 730 nm (OD_730_) and chlorophyll (Chl) content were determined using a spectrophotometer (DU 640, Beckman). For semi-continuous culturing under -P conditions, *Synechocystis* cells grown to the logarithmic phase under +P conditions were transferred to -P conditions and cultured for 36 h, maintaining the OD_730_ values of the culture between 0.1 and 0.5 through dilution with fresh BG11-P medium at specified intervals. Cells were harvested at specified times for DNA, RNA, or lipid isolation by centrifugation (3,000 rpm, 10 min). The OD_730_ value, and the chlorophyll (Chl) and phycobilisome (PBS) contents in the cultures were measured with a spectrophotometer (DU 640, Beckman, Hiyoshi et al., 2021).

### Isolation of DNA and RNA

DNA was isolated from *Synechocystis* cells with the use of NaI (Loparev et al., 1991). Harvested cells (less than 50 mg fresh weight) were resuspended in H_2_O and transferred to a microtube. The cells were pelleted by centrifugation (15,000 rpm, 10 min) and then resuspended in saturated NaI (200 μL) and left at 37°C for 30 min. The cell suspension was further resuspended with the addition of H_2_O (1 mL) and centrifuged (15,000 rpm, 2 min) to pellet the cells. This step was repeated once. The pellet was resuspended in a mixture (500 μL) containing 5 mg·mL^-1^ lysozyme, 50 mM Tris-HCl (pH 8.0), and 20 mM EDTA and incubated at 37°C for 30 min. The cells were then lysed through incubation for 2 h at 55°C after the addition of 10% SDS (50 μL) and 20 mg·mL^-1^ proteinase K (10.5 μL). DNA was extracted from the solution using phenol and a mixture of phenol and chloroform (1:1, vol.). The resultant DNA solution was treated with RNase, extracted with chloroform, and subjected to 2-propanol precipitation. DNA was dissolved in TE buffer and stored at -80°C until use.

RNA was isolated using a hot phenol extraction method (Aiba et al., 1981). Harvested cells of *Synechocystis* were resuspended at 65°C in a resuspension buffer containing 0.3 M sucrose, 10 mM sodium acetate, and 20 mM EDTA (125 μL) and lysed with a lysis buffer comprising 2% SDS and 10 mM sodium acetate (50 μL). The cell-lysis solution was transferred to a microtube and incubated at 65°C for 5 min after the addition of hot neutral phenol equilibrated with 100 mM Tris, pH 8.0 (50 μL), with vigorous shaking for 5 sec every other minute. The solution was incubated at 65°C for 5 min after the addition of acidic phenol equilibrated with H_2_O (250 μL), with vigorous shaking for 5 sec every other minute, left on ice for 5 min, and then centrifuged at 15,000 rpm for 5 min. The upper phase containing RNA with some DNA contamination was extracted with acidic phenol for RNA purification, which was repeated once. The recovered upper phase was extracted with a mixture of acidic phenol and chloroform (1:1, vol.), and finally with chloroform. The resultant solution was subjected to 2-propanol precipitation of RNA, followed by DNase I treatment. The DNase I-treated sample was subjected to RNA purification through phenol-chloroform extraction. RNA was precipitated with 2-propanol and dissolved in DEPC-treated H_2_O for storage at -80°C until use.

### Preparation and quantification of individual lipid classes

Total lipids were extracted from harvested cells of *Synechocystis* according to the method of Bligh and Dyer (1959). Total lipids were then separated into individual lipid classes by two-dimensional TLC for the measurement of their contents, as previously described (Sato et al., 2017a). The TLC plate was then sprayed with primulin and illuminated with UV light to detect separated lipid classes. Silica gels of individual lipid spots were scraped off and used for the preparation of methyl esters of their constituent fatty acids through treatment with 5% anhydrous methanolic HCl. The contents of fatty acid methyl esters in respective lipid classes were estimated by capillary GLC with arachidonic acid as an internal standard.

### Pi quantification in cells, RNA, DNA, and rRNA

Intact *Synechocystis* cells suspended in H_2_O, or DNA or RNA solution, were mixed with potassium peroxodisulfate solution (40 g·L^-1^) in a ratio of 5 to 1. The resultant solution was autoclaved at 120°C for 30 min to release Pi from P-compounds. Pi contents in respective samples were determined colorimetrically by quantifying the malachite green/phosphomolybdate complex, as previously described (Hiyoshi et al., 2021). Meanwhile, rRNA contents were estimated through agarose gel electrophoresis of RNA. rRNA in the gel was stained with ethidium bromide to obtain a fluorescent image, which was analyzed with ImageJ (http://rsbweb.nih.gov/ij/).

### Semi-quantitative and quantitative PCR analysis

Semi-quantitative reverse-transcriptase and quantitative real-time PCR (semi-qPCR and qPCR, respectively) analyses were performed as previously described by Sato et al. (2017b) using RNA prepared as described above. **Supplementary Table S1** shows primer sets for the amplification of *phoA*, *cdsA*, *pgsA*, *glpD*, and *plsX* sequences, respectively, for semi-qPCR analysis. RNA was also subjected to qPCR using a Rotor-Gene Q PCR System (Qiagen) with primer for the genes *glpD* and *plsX*, respectively (**Supplementary Table S2**). The expression levels of the respective genes were normalized to that of *rnpB*, a subunit of ribonuclease P, using primer sets in **Supplementary Tables S1** and **S2** in semi-qPCR and qPCR analyses, respectively.

## Results

### Quantitative changes of membrane lipids in *Synechocystis* cells under -P conditions

**Figure 1:**
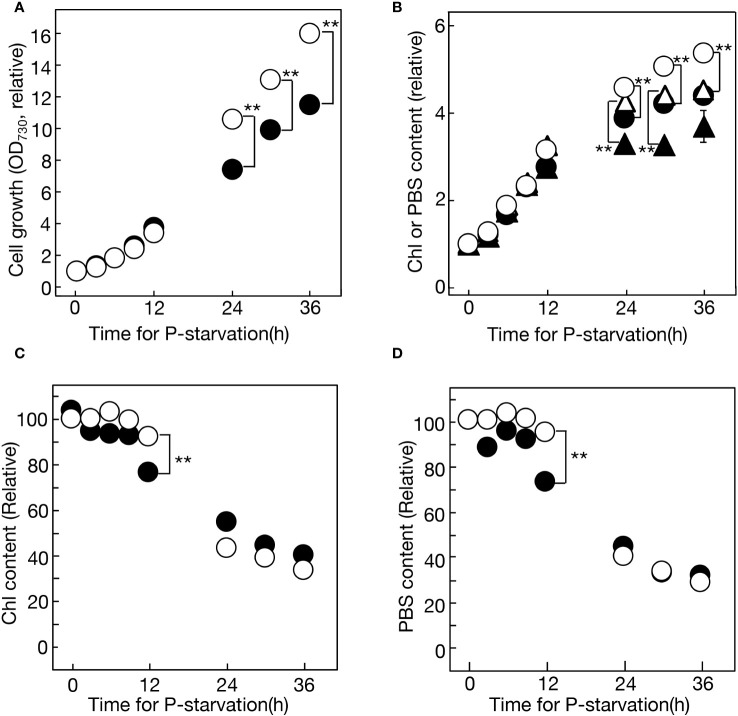
Cell growth and the accumulation of chlorophyll (Chl) and phycobilisomes (PBS) under -P conditions in semi-continuous or batch cultures of *Synechocystis* cells. **(A)** Cell growth, estimated based on OD_730_ values in the culture, relative to that at 0 h. The OD_730_ values at 0 h were 0.228 and 0.106 for semi-continuous and batch cultures, respectively. Open and closed circles represent semi-continuous and batch cultures, respectively. **(B)** Accumulation of Chl (circles) and PBS (triangles) in the culture, relative to that at 0 h. The Chl contents at 0 h were 1.41 and 0.677 μg·mL^-1^ for semi-continuous and batch cultures, respectively. The PBS contents at 0 h were 0.0587 and 0.0273 for semi-continuous and batch cultures, respectively. Open and closed symbols represent semi-continuous and batch cultures, respectively. Values in **(A, B)** in semi-continuous culture were estimated by multiplying them by the ratios of culture dilution with fresh BG11-P (**Supplementary Figure S1**). **(C)** Cellular Chl contents in semi-continuous cultures (open circles) and batch cultures (closed circles) were estimated by dividing the Chl content in **(B)** by OD_730_ values in **(A)**. **(D)** Cellular PBS contents in semi-continuous cultures (open circles) and batch cultures (closed circles) were estimated by dividing the PBS contents in **(B)** by OD_730_ values in **(A)**. The values in **(C)** or **(D)** are presented relative to that in +P cells at 0 h. The values shown are averages ± SE for three experiments. The significance of differences was evaluated by means of Student’s *t*-test. **P<0.01. Some error bars are hidden within symbols.

This study initially investigated lipid remodeling in *Synechocystis* cells responding to -P stress. Previous investigations of -P stress responses in photosynthetic microbes, including cyanobacteria, were primarily conducted in batch cultures. However, the batch culturing system inevitably involves nutrient shortages and shading as cell growth progresses. First, this study cultured *Synechocystis* cells semi-continuously, maintaining the OD_730_ values of the culture between 0.1 and 0.5 through dilution with fresh BG11-P medium (**Supplementary Figure S1**). This semi-continuous culturing system allowed for a focused examination of the impact of the sole stress of -P on cell physiology. Consistent with this approach, the semi-continuous -P culture of *Synechocystis* cells, in comparison to the batch culture, achieved higher levels of OD_730_, chlorophyll (Chl), and phycobilisomes (PBS) (**Figures 1A, B**). Moreover, when compared to batch culture, semi-continuous culture exhibited a delay in the decline of cellular Chl and PBS contents, i.e., a delay in the well-known symptoms of -P stress in cyanobacteria (**Figures 1C, D**; compare values at 12 h between the two culturing systems).

**Figure 2:**
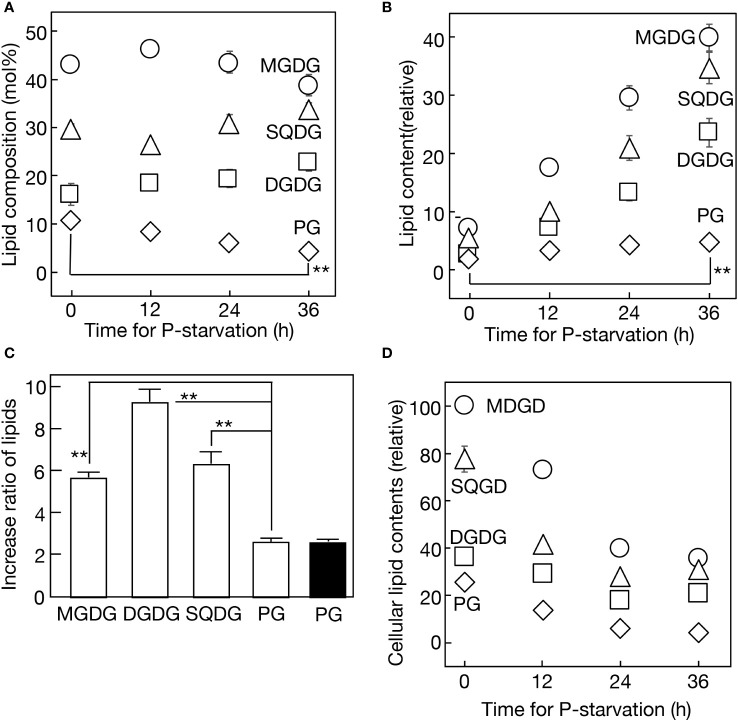
Quantitative changes in lipids in a semi-continuous culture of *Synechocystis* cells during -P. **(A)** Changes in lipid composition. **(B)** Accumulation of respective lipids in the culture. Respective lipid contents were estimated relative to the PG content at 0 h. **(C)** Lipid contents were respectively estimated after -P for 36 h, relative to their corresponding values at 0 h. White bars represent lipid contents in a semi-continuous culture, while a black bar represents the PG content in a batch culture. **(D)** Changes in cellular lipid contents were estimated by dividing the lipid contents in **(B)** by OD730 values, as represented in **Figure 1A**. The values are presented relative to that of MGDG at 0 h. The values shown are averages ± SE for three experiments. The significance of differences was evaluated by means of Student’s *t*-test. **P<0.01. Some error bars are hidden within symbols.

Next, four major membrane lipids were quantitatively analyzed in *Synechocystis* cells during 36 h of -P semi-continuous culturing (**Figure 2A**). PG decreased steadily from 10.9 to 4.6 mol% of total lipids, while SQDG increased from 29.8 to 33.7 mol%, with a minor dip at 12 h. Ultimately, *Synechocystis* exhibited the partial substitution of SQDG for PG, a phenomenon observed in various prokaryotic and eukaryotic photosynthetic organisms. Regarding galactolipids, monogalactosyl diacylglycerol (MGDG) experienced a slight decrease from 43.1 to 38.8 mol% over 36 h, with a minor peak at 12 h, while digalactosyl diacylglycerol (DGDG) increased steadily from 16.1 to 22.8 mol%. The analysis of lipid classes in the culture revealed constant increases under -P conditions, with DGDG exhibiting the most rapid increase (9.3-fold in 36 h), followed by SQDG (7.4-fold), MGDG (5.9-fold), and PG (2.7-fold) (**Figures 2B, C**). The steady increase in PG in the -P culture indicated the preferential synthesis of PG among P compounds. The decrease in PG relative to total lipids was thus attributed to its dilution by the more rapid increases in glycolipids. Meanwhile, similar to cellular Chl and PBS contents, the respective contents of cellular lipids decreased over time (**Figure 2D**). It was noteworthy that the cellular lipid decrease occurred even in the first 12 h, despite a simultaneous little decrease in cellular Chl or PBS (see semi-continuous culture in **Figures 1C, D**). This suggested that the reduction in lipid content in thylakoid membranes preceded that in the size of the photosynthetic machinery during the early phase of -P stress.

### Regulatory expression of the genes responsible for PG synthesis in *Synechocystis* cells under -P conditions

**Figure 3:**
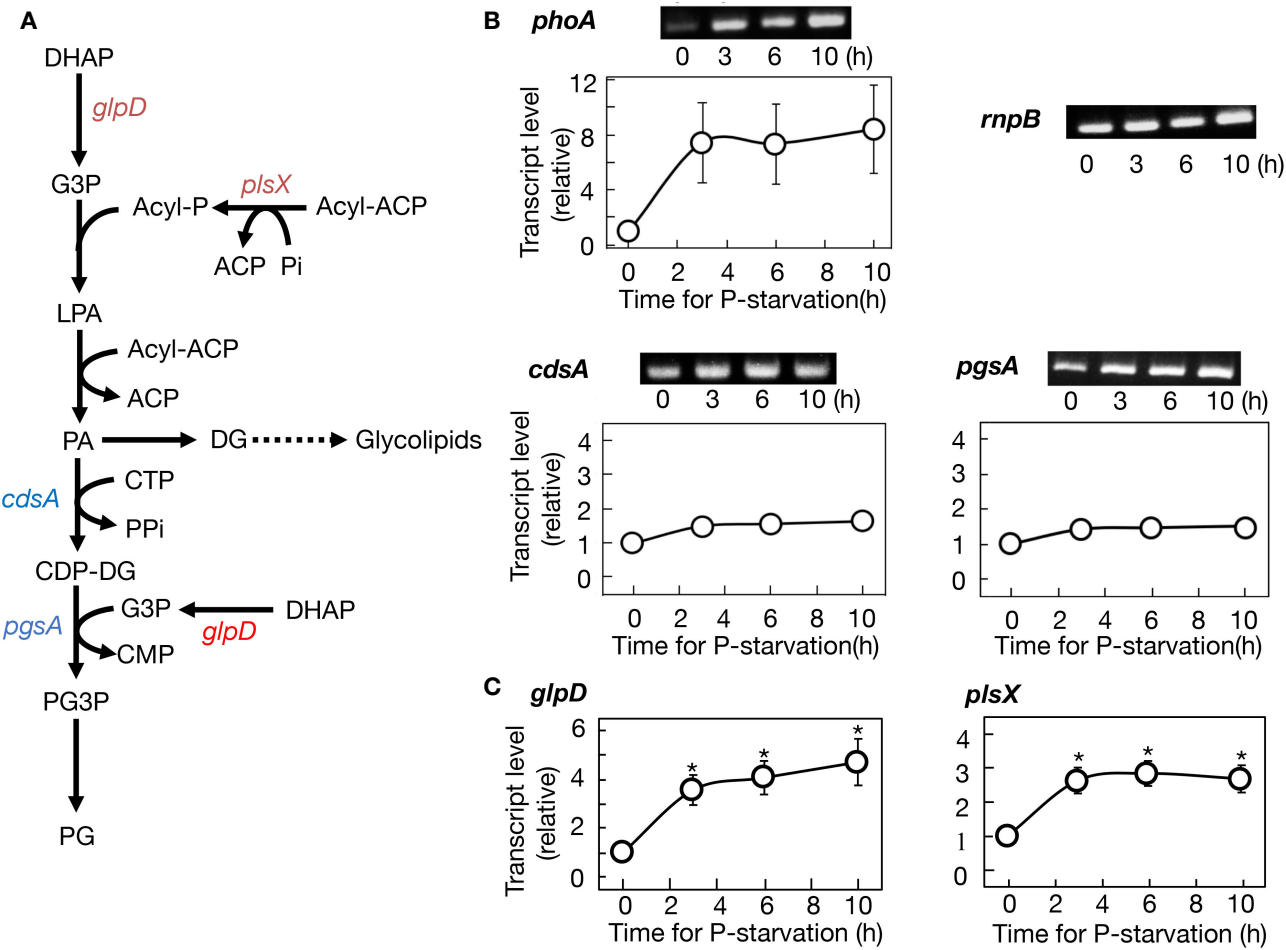
Regulation of gene expression for PG synthesis promotion in *Synechocystis* cells under -P conditions. **(A)** The metabolic map of PG synthesis. Genes upregulated at their transcript levels and those little altered at the levels are shown in red and blue, respectively. **(B)** Semiquantitative PCR analysis of *phoA* encoding alkaline phosphatase, *cdsA*, and *pgsA*. Transcript levels were estimated relative to those of *rnpB*, as described in Materials and Methods. Note that the expression level of *phoA* was markedly increased under -P conditions, as previously reported (Suzuki et al., 2004). **(C)** Quantitative PCR analysis of *glpD* and *plsX*. The values shown are averages ± SE for three experiments. The significance of differences at 3, 6, or 10 h relative to at 0 h was evaluated by means of Student’s *t*-test. *P<0.05. Some error bars are hidden within symbols.

The facilitation of PG synthesis in -P cells, our key finding concerning the well-conserved SQDG substitution for PG, was also observed in the batch culture (**Figure 2C**). The batch culturing system, due to its simplicity, was then utilized to investigate the molecular mechanism behind this facilitation. Two genes, *cdsA* and *pgsA*, which encode CDP-diacylglycerol and phosphatidylglycerolphosphate synthases, respectively, participate in the formation of the polar head group of PG (**Figure 3A**). Semi-qPCR analysis revealed little change in the mRNA levels of *cdsA* or *pgsA* for at least 10 h under -P conditions, in contrast to the marked upregulation of *phoA* mRNA, as previously reported by Suzuki et al. (2004) (**Figure 3B**). The *glpD* gene, which encodes glycerol 3-phosphate dehydrogenase responsible for converting dihydroxyacetone phosphate (DHAP) to glycerol 3-phosphate. Glycerol 3-phosphate is essential for glycerolipid synthesis, and notably crucial for the formation of the polar head group of PG, as depicted in **Figure 3A**. Semi-qPCR analysis demonstrated that *glpD* was upregulated at the mRNA level under -P conditions (**Supplementary Figure S2**). Meanwhile, *plsX*, encoding phosphate acyltransferase and utilizing Pi for acyl-phosphate synthesis in glycerolipid synthesis, displayed a similar upregulation pattern to that of *glpD* (**Supplementary Figure S2**). Furthermore, it was confirmed that *glpD* and *plsX* were maximally upregulated by 4.3- and 3.0-fold, respectively, through qPCR (**Figure 3C**). In a situation where P-containing DHAP and Pi are limited in -P cells, the enhanced expression of *glpD* and *plsX* would contribute to their metabolic flow into glycerolipid synthesis, particularly PG synthesis.

### Intracellular Pi-source for preferential synthesis of PG in *Synechocystis* cells under -P conditions

**Figure 4:**
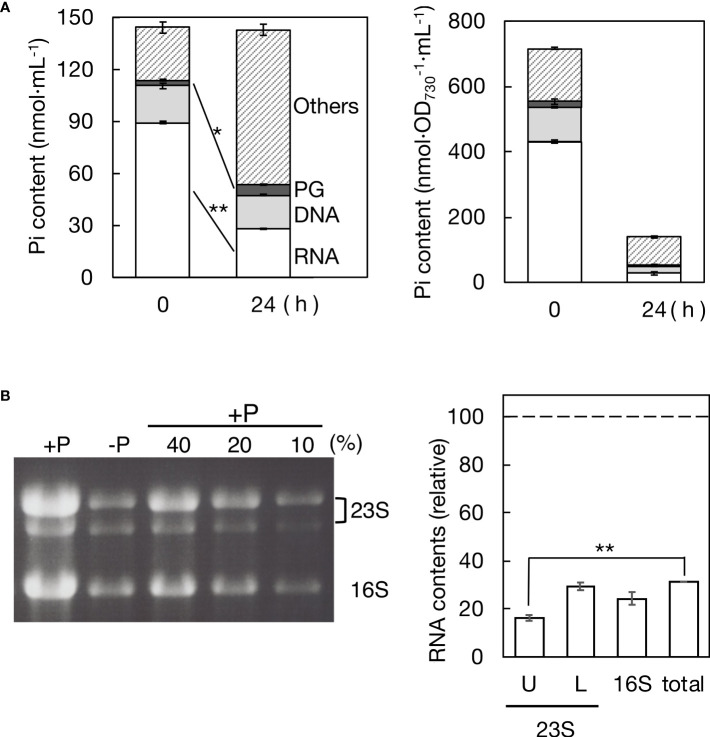
Remobilization of phosphorus (P) in a batch culture of *Synechocystis* cells under -P conditions. **(A)** Pi distribution to RNA, DNA, PG, and the rest (referred to as the other P-compounds) in +P or -P cells. Pi contents of cells, RNA, and DNA were estimated through calorimetric analysis of Pi, whereas Pi content of PG was estimated through fatty acid analysis by GC. Left panel: Pi contents in a culture of +P cells at OD_730_ = 0.2 (0 h) and those in a batch culture where these cells were grown for 24 h after the shift to -P conditions (24 h). Right panel: cellular Pi contents in +P (0 h) and -P cultures (24 h) estimated by dividing the data in the left panel by OD_730_ values of the cultures (0.20 and 0.94 ± 0.05 at 0 and 24 h, respectively). **(B)** Total RNA isolated from +P and -P cultures was utilized for rRNA analysis through agarose gel electrophoresis. Left panel: photograph of the gel, where 23S and 16S rRNAs were stained with ethidium bromide. Three lanes labeled with 10, 20, and 40% indicate the proportions of RNA samples from the +P culture used to create standard curves. Right panel: contents of respective rRNA species and total cellular RNA in -P culture, relative to their corresponding ones in +P culture. U, L, and 16S indicate the upper and lower bands of 23S rRNA and 16S rRNA while ‘total’ indicates total cellular RNA. The contents of 23S and 16S rRNA species were determined by measuring their fluorescence intensities, as described in Materials and Methods, followed by quantification using standard curves. The total cellular RNA content was estimated based on the data in **(A)**. The values shown are averages ± SE for three experiments. The significance of differences was evaluated by means of Student’s *t*-test. **P<0.01. *P<0.05.

The quantitative behavior of P in nucleic acids, which typically constitute the majority of cellular P compounds, was investigated in *Synechocystis* cells following the transfer from +P to -P conditions (see the left panel of **Figure 4A**). Cells in culture retained 99.2% of the initial total P after 24 h of exposure to -P stress (142.9 μmol Pi·L^-1^ compared to 144.2 μmol Pi·L^-1^ at 0 h), indicating minimal leakage of Pi from the cells. In +P culture, RNA represented 89.4 μmol Pi·L^-1^ (62.0% of total Pi), which drastically decreased to 28.1 μmol Pi·L^-1^ (19.7% of total Pi) within 24 h under -P conditions. DNA accounted for 21.0 μmol Pi·L^-1^ (14.6% of total Pi) in +P culture, only slightly decreasing to 19.6 μmol Pi·L^-1^ (13.7% of total Pi) in -P culture. The increased Pi content in PG from 3.4 to 5.9 μmol Pi·L^-1^ (2.4 to 4.1% of total Pi) aligned with the results in **Figures 2B, C**. Meanwhile, the Pi content in a fraction of the other P-compounds ("Others" in **Figure 4A**) increased from 30.3 to 89.3 μmol Pi·L^-1^ (21.0 to 62.5% of total Pi). Collectively, RNA underwent significant degradation, decreasing to as low as 31.4% of the initial level (see the right panel of **Figure 4B**), in contrast to relatively stable DNA. Notably, the decreased Pi content in RNA (61.3 μmol Pi·L^-1^) was nearly equivalent to the increased Pi content in PG + others (61.5 μmol Pi·L^-1^), highlighting the significant role of RNA as an internal Pi supplier for the synthesis of these P compounds.

Cellular-P decreases were estimated in nmol P·OD_730_^-1^·mL^-1^ as follows (see the right panel of **Figure 4A**): from 716.6 to 138.7 in total P (i.e., to 19.4% of the initial level), from 431.0 to 27.2 (6.3%) in RNA, from 105.2 to 21.0 in DNA (20.0%), from 17.0 to 6.1 (35.9%) in PG, and from 163.5 to 84.4 (51.6%) in the other P-compounds. Concerning the retention ratio, DNA was almost equal to total P, which suggested that the cellular decrease in DNA was mainly due to cell division, i.e., DNA transfer to daughter cells. Meanwhile, PG was 1.8-fold higher than total P in the retention ratio, highlighting the physiological significance of PG in *Synechocystis* cells acclimating to -P stress.

rRNA, which generally predominates bacterial cellular RNA (more than 80%, Planson et al., 2020), was then measured in the culture by agarose gel electrophoresis of total RNA (**Figure 4B**). 23S rRNA species appeared as two bands in the gel (Mironov & Los, 2015), with degradation to 16.3% and 29.4% in the upper and lower bands, respectively, after -P for 24 h, relative to the initial levels. Additionally, 16S rRNA exhibited degradation to 24.4%. rRNA degradation was more pronounced than that in total RNA (31.4%). These results demonstrate that rRNA plays a major role as an internal-P source for P remobilization in *Synechocystis* cells.

## Discussion

### Promotion of remobilization of internal P into PG synthesis in *Synechocystis* under -P conditions

Membrane lipid remodeling is a crucial strategy for photosynthetic organisms to acclimate to environmental stress conditions (Yu et al., 2021). Specifically, the replacement of PG by SQDG in response to -P stress appears to be a conserved mechanism across prokaryotic and eukaryotic photosynthetic organisms (Güler et al., 1996; Yu et al., 2002; Riekhof et al., 2003; Sato and Wada, 2009; Van Mooy et al., 2009; Endo et al., 2016; Murakami et al., 2018; Oishi et al., 2022). However, it has remained unclear whether -P stress alone is sufficient to induce PG-SQDG remodeling. This study demonstrated that the sole -P stress indeed triggers this lipid remodeling in *Synechocystis* with the use of a semi-continuous culturing system, and uncovered the metabolic mechanisms underlying this phenomenon (**Figure 2**). Firstly, PG was preferentially synthesized among cellular P-compounds under -P conditions (**Figures 2B, C**). The increased metabolic flow of P into PG in -P cells, relative to +P cells, was previously suggested in a batch culture of another cyanobacterium, *Thermosynechococcus* elongatus BP-1 (Endo et al., 2016). Our detailed time-course experiments provided clear evidence that the sole -P stress induces the continuous remobilization of internal P into PG synthesis in *Synechocystis* cells, leading to its increase of up to more than twofold in the culture. Secondly, despite the stimulated PG synthesis, its content in total lipids decreased due to dilution by glycolipids, which were synthesized at much faster rates (**Figures 2B, C**). This phenomenon resulted in the partial replacement of PG by SQDG, improving PUE. Thirdly, the active synthesis of PG seemed to be achieved through the elevated expression of *glpD* and *plsX* genes involved in construction of the diacylglycerol moiety of membrane lipids, including PG, with *plsX* contributing also to the formation of its polar head group (**Figure 3C**). Protein amounts of GlpD and PlsX, together with their cellular activities, will be studied in the future. In contrast, the *cdsA* and *pgsA* genes, which are responsible specifically for PG synthesis, showed almost no elevation in mRNA levels (**Figure 3B**), suggesting that their mRNA levels in +P cells were already high enough to promote PG synthesis after shifting to -P conditions. Intriguingly, it appeared that both *cdsA* and *pgsA* mRNAs were present at excessively high levels in +P cells of *Thermosynechococcus* elongatus BP-1 since these mRNA levels were little altered in its SQDG-deficient mutant that elevated the PG content (Endo et al., 2016).

Cyanobacteria, including *Synechocystis*, accumulate glycogen and/or poly-β-hydroxybutyrate (PHB) under -P conditions (Kromkamp, 1987; Monshupanee and Incharoensakdi, 2014; Kaewbai-Ngam et al., 2016; Hirai et al., 2019). The synthesis of these C-storage compounds consumes surplus chemical energy, such as ATP, under -P conditions, thereby preventing the overreduction of the photosynthetic electron transport chain, and accordingly, the production of toxic reactive oxygen species (Ghirardi et al., 2018). Dihydroxyacetone phosphate (DHAP) and Pi are essential for glycogen and PHB synthesis, serving as a fixed carbon supplier and a substrate for ATP synthesis, respectively. The upregulation of *glpD* and *plsX* induced by -P supports the diversion of DHAP and Pi into PG synthesis, in competition with active glycogen and PHB synthesis. In the future, the metabolic and molecular mechanisms of -P-induced PG decrease in total lipids, including the possibility of stimulation of PG synthesis as observed in *Synechocystis*, will be examined in other cyanobacteria, algae, and seed plants.

When algae and seed plants are exposed to -P conditions, they undergo a different type of lipid remodeling in extraplastid membranes (Nakamura, 2013). This involves the degradation of PC and PE, along with compensatory increases in non-phospholipid contents in species-dependent manners, i.e., with an increase in DGDG in *A. thaiana* and an increase in a betaine lipid, diacylglyceryl-*N*,*N*,*N*-trimethylhomoserine, in a green alga, *Chlorella kessleri* (Härtel et al., 2000; Oishi et al., 2022). Notably, in *C. kessleri*, both PC and PE become undetectable in -P cells, being completely replaced by DGTS (Oishi et al., 2022). It is essential to emphasize that unlike PC and PE, PG does not undergo significant degradation in *Synechocystis* under -P conditions. Intriguingly, in green algae, SQDG, which constitutes 5-11% of total cellular sulfur (S), is almost entirely degraded in chloroplast membranes under sulfate-starvation conditions, with a simultaneous increase in PG (Sugimoto et al., 2007; Sugimoto et al., 2010; Sato et al., 2017a). This almost complete replacement of SQDG by PG seems to be established owing to the non-essential roles of SQDG in green algal cell growth (Sato et al., 1995a; Sato et al., 1995b; Sato, 2004). In contrast to SQDG in green algae, PG is essential for cell growth in cyanobacteria due to its involvement in various critical processes in cell membranes, including chlorophyll synthesis, efficient electron transfer from Q_A_ to Q_B_, PSI and PSII construction, and cell division (Sato et al., 2000; Sato et al., 2004; Mizusawa and Wada, 2012; Kopečná et al., 2015; Kóbori et al., 2018). The -P-induced promotion of PG synthesis in *Synechocystis* may be necessary to provide PG molecules for -P acclimation processes. Future research should aim to further investigate this concept, including the identification of PG-demanding physiological processes in -P *Synechocystis* cells.

### Intracellular P-source in *Synechocystis*

This study, through the quantification of inorganic phosphate (Pi) in major cellular P-compounds, specifically nucleic acids, demonstrated that rRNA degradation serves as a major intracellular-P source for P remobilization in *Synechocystis* under -P conditions (**Figure 4**). The degradation of rRNA likely reflected the reduced need for ribosomes in -P cells, where metabolic activities slowed down. Another potential intracellular-P source is polyphosphate, which is degraded under -P conditions in various bacteria, including *Synechocystis* (Li and Dittrich, 2019; Hiyoshi et al., 2021). However, Pi in polyphosphate accounted for only around 10% of total cellular P under normal conditions in *Synechocystis*, which was much smaller than the fraction of degraded rRNA (42.9%). Thus, we propose that under -P conditions, *Synechocystis* cells utilize rRNA as the sole major intracellular-P source for the synthesis of PG and other P-compounds that are yet to be identified.

The limited DNA degradation observed in -P culture of *Synechocystis* cells suggested that the reduction in cellular DNA content, i.e., genomic DNA copy number, primarily occurs through cell division (**Figure 4**). Consistent with this idea, a subset of *Synechocystis* strains was reported to decrease genome copy numbers under -P stress (Zerulla et al., 2016). In contrast, DNA in the chloroplast, which is the presumed descendant of endosymbiotic cyanobacteria, was positively degraded in -P cells of *Chlamydomonas reinhartdtii* to expand the internal-P pool (Yehudai-Resheff et al., 2007). Chloroplast DNA has only a restricted set of genes in accordance with its small size in *C. reinhardtii* (203 kbp; Maul et al., 2002) compared to chromosomal DNA in *Synechocystis* (3573 kbp; Kaneko and Tabata, 1997). Furthermore, chloroplast DNA typically had 80 copies in a *C. reinhardtii* cell, whereas chromosomal DNA in *Synechocystis* was present at most with 14 copies (Zerulla et al., 2016; Gallaher et al., 2018). These properties of chloroplast DNA may enable *C. reinhardtii* cells to utilize it as an internal-P source. On the contrary, chromosomal DNA appears to be spared from degradation in -P *Synechocystis* cells, given its extensive genome coverage and low copy number. Future studies would involve investigating whether other photosynthetic microbes utilize rRNA as the primary internal-P source for P-remobilization under -P conditions.

## Data availability statement

The original contributions presented in the study are included in the article/**Supplementary Material**, further inquiries can be directed to the corresponding author.

## Author contributions

TH: Investigation, Methodology, Validation, Writing – review & editing. MH: Investigation, Methodology, Validation, Writing – review & editing. NS: Investigation, Methodology, Validation, Writing – review & editing, Conceptualization, Funding acquisition, Supervision, Writing – original draft.
