# Supplementary material for: Preferential phosphatidylglycerol synthesis via phosphorus supply through rRNA degradation in the cyanobacterium, Synechocystis sp. PCC 6803, under phosphate-starved conditions
Source: Front Plant Sci. 2024 Jan 29;15:1335085. doi: 10.3389/fpls.2024.1335085 (PMC10859501; doi:10.3389/fpls.2024.1335085)

Table S1 Primer sets for semi-qPCR analysis

Forward Reverse

*phoA* 5'GTTTCAGTCCTGTGCCCAAC 3' 5' TGCACTTGACCAGCTTCTTG 3'

*cdsA* 5' ACTGGGCCTGTTTTATGGGG 3' 5' GAAAACGTAGCTGTCGGTGC 3'

*pgsA* 5' CTGTTTCCCGTTTATTGGCT 3' 5' TCGACTTTGTTGTTTCCCGG 3'

*glpD* 5' ACAGTTCCAGCAACTGTTCC 3' 5' CATCACTGGCTTTAACCCTG 3'

*plsX* 5' GAAGGGGTGGTGGAAATGGA 3' 5' AAGTACGTCCCTTCCTTCGG 3'

*rnpB* 5' AAGAGCGCACCAGCAGTATC 3' 5' TAAGCCGGGTTCTGTTCC 3'

Table S2 Primer sets for qPCR analysis

Forward Reverse

*glpD* 5' GTGGGAGAAGAAATGGTGGAT 3' 5' CGGTAAAGGTTGGGTCAGG 3'

*plsX* 5'GGCACCTTATTTCCCACAATG3' 5'GGCTATCAACCCCCAACAC3'

*rnpB* 5'AAAGGGTAAGGGTGCAAAGG3' 5'AATTCCTCAAGCGGTTCCAC3'


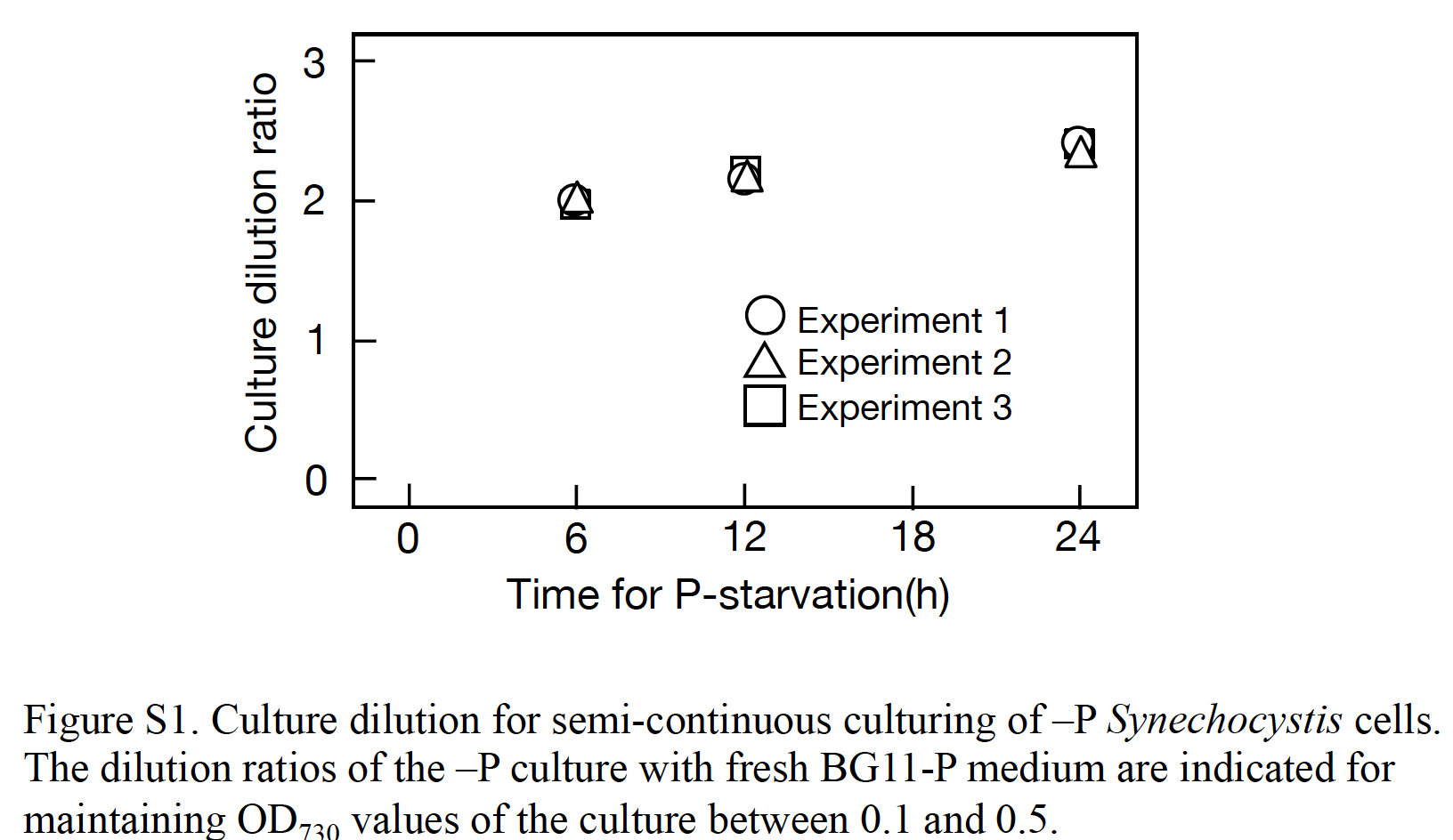


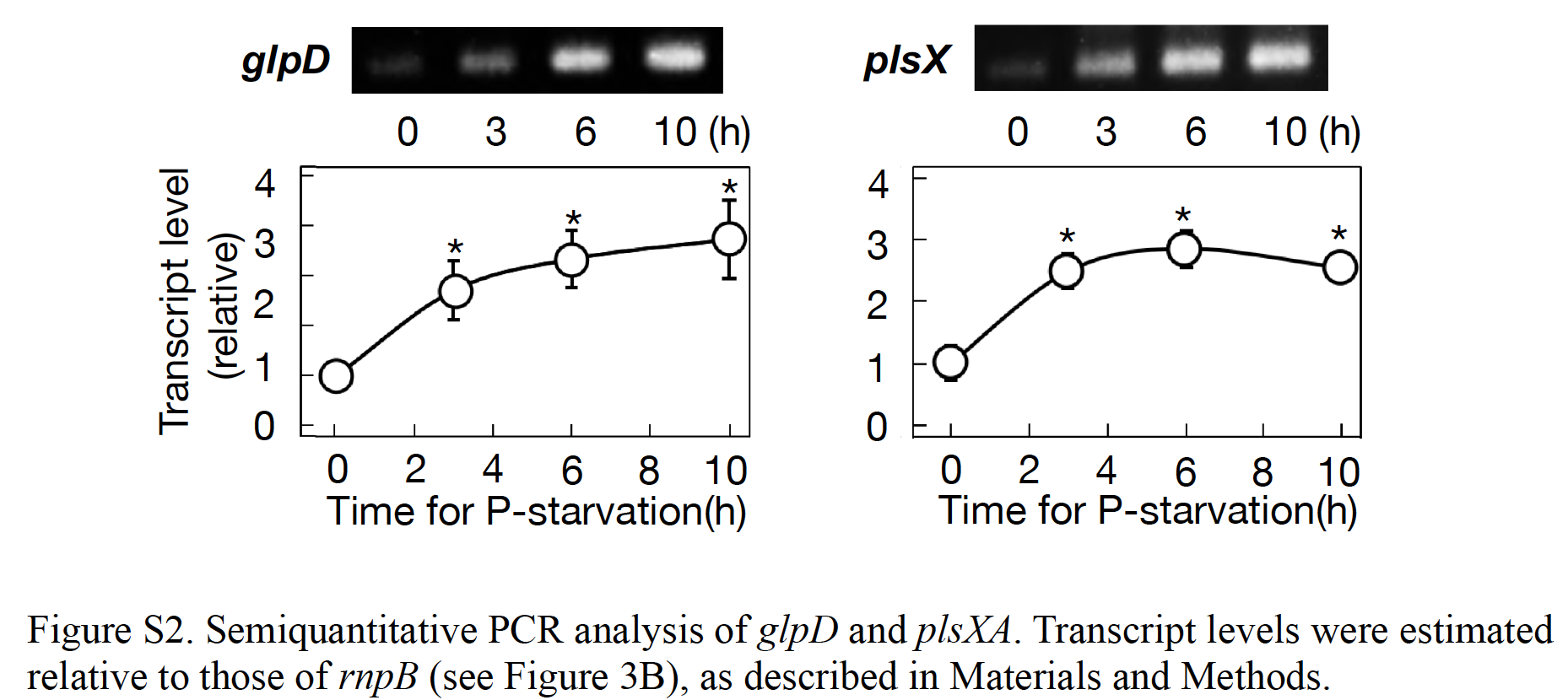

Supplement: Supplementary file 1 [file DataSheet_1.docx]
